# Alterations of protein expression in conditions of copper-deprivation for *Paracoccidioides lutzii* in the presence of extracellular matrix components

**DOI:** 10.1186/s12866-014-0302-7

**Published:** 2014-12-13

**Authors:** Haroldo Cesar de Oliveira, Julhiany de Fátima da Silva, Marcelo Teruyuki Matsumoto, Caroline Maria Marcos, Roberta Peres da Silva, Rosângela Aparecida Moraes da Silva, Mônica Teresa Veneziano Labate, Carlos Alberto Labate, Ana Marisa Fusco Almeida, Maria José Soares Mendes Giannini

**Affiliations:** Departamento de Análises Clínicas, Faculdade de Ciências Farmacêuticas, UNESP - Univ Estadual Paulista, Laboratório de Micologia Clinica, Rodovia Araraquara-Jaú, Km 1, Araraquara, SP Brazil; Departamento de Genética, Escola Superior de Agricultura “Luiz de Queiroz, Universidade de São Paulo, Laboratório Multiusuários Centralizado de Genômica Funcional Aplicada à Agropecuária e Agroenergia, Piracicaba, São Paulo Brazil

**Keywords:** *Paracoccidioides* spp, Copper, Adhesion, Protein expression, Paracoccidioidomycosis

## Abstract

**Background:**

*Paracoccidioides* spp is a fungi genus and the agent of paracoccidioidomycosis. The strategies of infection used by these pathogens involve the expression of proteins related to adaptation to the host, particularly regarding the uptake of micronutrients. This study analyzed the adhesion of *Paracoccidioides lutzii* during conditions of copper (Cu) and iron (Fe) deprivation, while also evaluating the proteins expressed in conditions of Cu depletion in the presence of four extracellular matrix (ECM) components (laminin, fibronectin and types I and IV collagen).

**Results:**

We cultured the *P. lutzii* in a chemically defined media without Cu and Fe. The fungus was then placed in contact with different ECM components and adhesion was evaluated. A significant increase in binding to all ECM components was observed when the fungus was cultured without Cu; which might be related to some adhesins expression. A proteomic assay was developed and revealed 39 proteins expressed that are involved in processes such as virulence, protein synthesis, metabolism, energy, transcription, transport, stress response and the cell cycle when the fungus was interacting with the ECM components. The up-regulated expression of two important adhesins, enolase and 14-3-3, was observed at the fungal cell wall during the interaction with the ECM components, indicating the role of these proteins in the *Paracoccidioides*–host interaction.

**Conclusions:**

This study is important for determining prospective proteins that may be involved in the interaction of *Paracoccidioides* with a host. Understanding the adaptive response to different growth conditions, elucidating the processes of adhesion and cell invasion, and identifying the proteins that are differentially expressed during the fungus-host interaction may help elucidate mechanisms used for survival and growth of *Paracoccidioides* in various human tissues.

**Electronic supplementary material:**

The online version of this article (doi:10.1186/s12866-014-0302-7) contains supplementary material, which is available to authorized users.

## Background

*Paracoccidioides lutzii,* a specie from a complex genus [1-4], are dimorphic fungi and the etiologic agents of paracoccidioidomycosis (PCM), which is the most important systemic mycosis in Latin America [5]. *Paracoccidioides* is a notably versatile pathogen, with the ability of infecting numerous systems and organs of the human body, because it has developed mechanisms that enable adherence and invasion of host tissues [6].

The *Paracoccidioides* species initiates host infection by adhering to components of the extracellular matrix (ECM); this adherence is mediated by a variety of adhesins on the fungal surface, with component recognition of adhesive matrix molecules, which plays an important role in the regulation of cell adhesion, differentiation, migration and proliferation [7]. Some *Paracoccidioides* adhesins have been described and are included in microorganism strategies of evading the immune system and ensuring survival in hosts. Adhesion is closely associated with the transcriptional control of several regulatory pathways that control the synthesis of these molecules. These pathways are activated in response to various conditions, such as nutrient limitation [8], which is vital for any pathogen.

To successfully colonize a host, *Paracoccidioides* must initially adhere to host tissues and simultaneously obtain essential nutrients for growth and survival. Iron (Fe) and copper (Cu) are required for survival, primarily due to their roles as cofactors for many essential metabolic functions. Cu is an essential micronutrient for all biological systems, with multiple proteins requiring one or more atoms of Cu to achieve the appropriate structure and function. To prevent the consequences of Cu deficiency, living organisms have evolved molecular mechanisms that regulate the uptake, intracellular traffic, storage and efflux of Cu. Some of the cellular responses to variations in Cu levels are related to changes in the expression of genes encoding the molecular components of Cu metabolism. Cu serves as a catalytic and structural cofactor for enzymes involved many processes, including energy generation, Fe acquisition, oxygen transport and cellular metabolism [9]. Both the host and fungi have developed sophisticated strategies for acquiring the metals, even under conditions of limited availability. Several homeostatic mechanisms have been demonstrated in fungi, guaranteeing the maintenance of sufficient concentrations of Cu for cell growth without causing damage. Additionally, posttranslational mechanisms, such as the intracellular trafficking of Cu transporters, have been identified in mammals. In these organisms, Cu homeostasis is also mediated by the transcriptional regulation of genes involved in Cu acquisition, mobilization, and sequestration [10,11].

During the infection process, the levels of free Fe and Cu are significantly reduced; the acquisition of these nutrients is related to a higher adaptive process that is important for microorganism virulence, as demonstrated in several organisms. In *Candida albicans,* increased gene expression was observed during Fe privation related to virulence, such as secreted hydrolase genes [12]. Similarly, mutations in the genes involved in Fe capture, such as the Fe oxidase gene in *C. albicans* [13] and the Fur gene from *Helicobacter pylori* [14], rendered these microorganisms incapable of colonizing host tissues and causing infection. In *Cryptococcus neoformans,* the increased expression of Cu transporters is related to the dissemination of the species in the host meninges [15]. In *Paracoccidioides*, low Fe conditions have been associated with fungal susceptibility to the antimicrobial action of monocytes [16]. The administration of exogenous Fe results in an increased fungal load in mouse tissues infected with *Paracoccidioides* [17].

Proteomic methods have been used to study the biology of *Paracoccidioides.* Historically, studies have focused on dimorphism and the characterization of single or a few protein targets in *Paracoccidioides* species [18-20], with the obtained information constituting a useful resource for studying the dimorphism of *Paracoccidioides* [21]. Parente *et al.* [17] used 2-DE to identify proteins related to *Paracoccidioides* survival in a Fe-deficient environment. They discovered that, during Fe starvation, fungi use the glycolytic pathway to obtain energy instead of the oxidative pathway, which is dependent on enzymes containing Fe-S groups. In addition, those authors identified activation of the Fe uptake systems as an indispensable survival mechanism required by the fungus in this type of environment [17]. Vallejo *et al.* [22] studied the secretome of *Paracoccidioides* with regards to the fungal extracellular vesicles that are able to cross the cell wall and transport molecules that facilitate nutrient acquisition, cell defense, and modulation of the host defense machinery. In another study, those authors determined that *Paracoccidioides* shared secreted proteins among *Histoplasma capsulatum, C. neoformans* and *Saccharomyces cerevisiae* [23]. The mycelia and yeast cell secretomes of *Paracoccidioides* have also revealed that many proteins do not use the classical secretory pathway, while many other proteins likely exerted other activities, once secreted. *Paracoccidioides* data has indicated that it uses non-classical targeting mechanisms to direct protein export and also that it has molecules that can function as virulence factors [24]. De Arruda Grossklaus *et al.* [25] also studied the oxidative stress response of *Paracoccidioides* using proteomic analysis.

Our aim was to analyze the adhesion of *Paracoccidioides lutzii* (Pb01 isolate) in Cu and Fe depleted conditions, while also determining the proteins that may be involved in the interaction among *Paracoccidioides* and ECM components that lead to an increase in fungal adhesion when the fungus was deprived of Cu. These proteins are involved in distinct biological processes, such as the cell cycle, stress response and Fe transportation; additionally, several proteins have been described as virulence factors and possible candidates for *Paracoccidioides* virulence. The results of this study provide new knowledge that may facilitate strategies used by *Paracoccidioides* to successfully parasitize host tissues, leading to the development of paracoccidioidomycosis.

## Results

### Adhesion of *Paracoccidioides lutzii* to ECM components during Cu and Fe depletion

**Figure 1:**
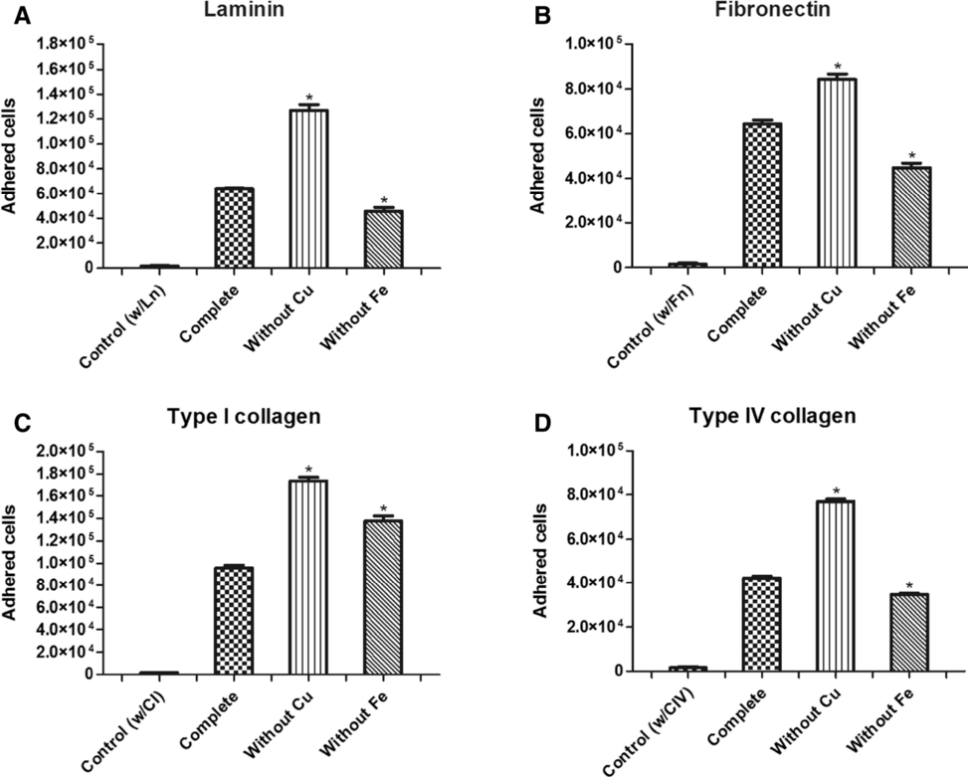
***P. lutzii*** **adhesion to ECM components laminin (A), fibronectin (B), type I collagen (C), and type IV collagen (D) with or without Cu and Fe.** *p < 0.05. These data represent numbers of three independent experiments, in triplicate. The first bars in all graphs show the experiment control where the fungal suspension was inoculated in wells without ECM components.

Following incubation in MVM without Cu (MVM-W-Cu medium), the *P. lutzii* yeast phase presented a significant increase (p < 0.05) in adhesion (Figure 1) in the presence of all tested ECM components (laminin, fibronectin, and types I and IV collagen), while in MVM without Fe (MVM-W-Fe), we observed a significant decrease (p < 0.05) in adhesion in the presence of laminin, fibronectin and type IV collagen and an increase in the presence of type I collagen, but this increase was significantly lower (p < 0.05) than the increase observed under Cu depletion.

### Adhesin expression profile during Cu and Fe depletion in the presence of ECM components

**Figure 2:**
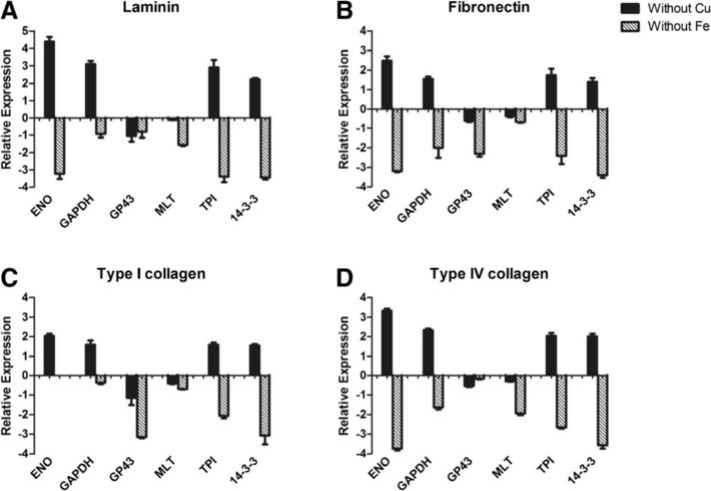
***P. lutzii*** **adhesins expression profile during interaction with laminin (A), fibronectin (B), collagen type I (C), and collagen type IV (D) after Cu and Fe depletion.**

The expression of genes that encoded most of the known *Paracoccidioides* adhesins during the interaction with the host ECM components (laminin, fibronectin and types I and IV collagen) were analyzed. We used real-time PCR to determine whether the metal depletion modulated adhesin expression. As shown in Figure 2, we observed that different micronutrient depletion conditions led to different levels of adhesin expression.

Cu depletion led to increased expression of the main adhesins, except for gp43 and malate synthase (MLT), whereas Fe depletion led to decreased expression in all studied adhesins. These effects corroborate the results of the adhesion experiments and revealed the importance of these proteins to the *Paracoccidioides*-host interaction, in which increased and decreased adhesion may be related to adhesin expression.

### Immunogold expression analysis of enolase and 14-3-3 proteins in *P. lutzii* yeast cells during interaction with ECM components after micronutrient depletion

**Figure 3:**
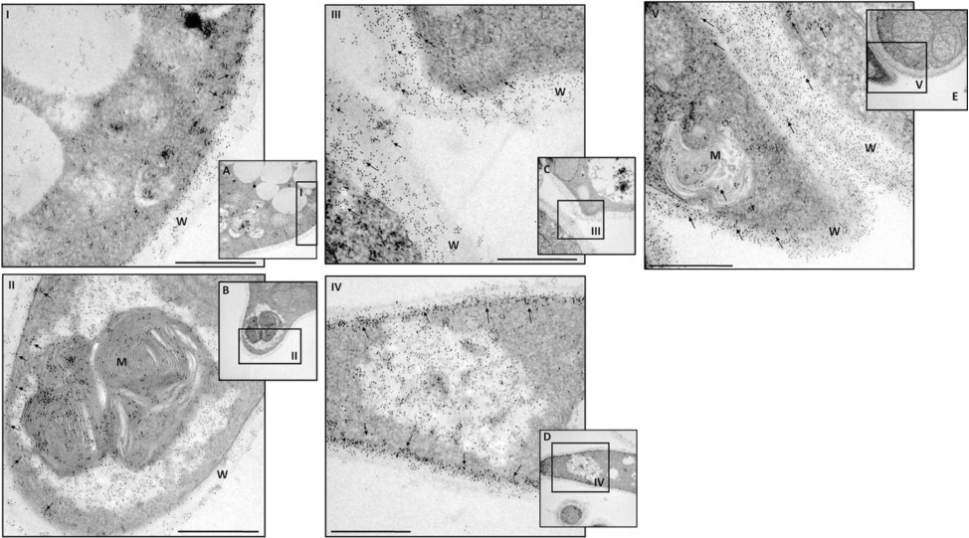
**IEM detection of enolase in** ***P. lutzii*** **yeast cells without Cu (A), and without Cu add laminin (B), fibronectin (C), type I collagen (D), or type IV collagen (E).** The arrows indicate enolase labeled with gold particles. Bars: 0.07 μm. W: cell wall; M: mitochondria; V: intracellular vesicles. I, II, III, IV and V indicate which image region has been increased in the microscope.

**Figure 4:**
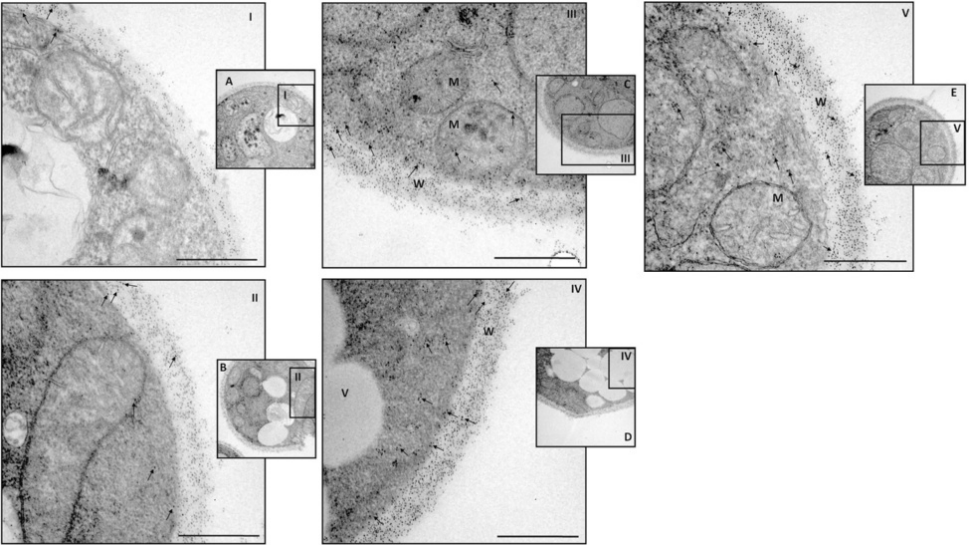
**IEM detection of 14-3-3 protein in** ***P. lutzii*** **yeast cells without Cu (A), and without Cu add laminin (B), fibronectin (C), type I collagen (D), or type IV collagen (E).** The arrows indicate 14-3-3 labeled with gold particles. Bars: 0.07 μm. W: cell wall; M: mitochondria; V: intracellular vesicles. I, II, III, IV and V indicate which image region has been increased in the microscope.

**Figure 5:**
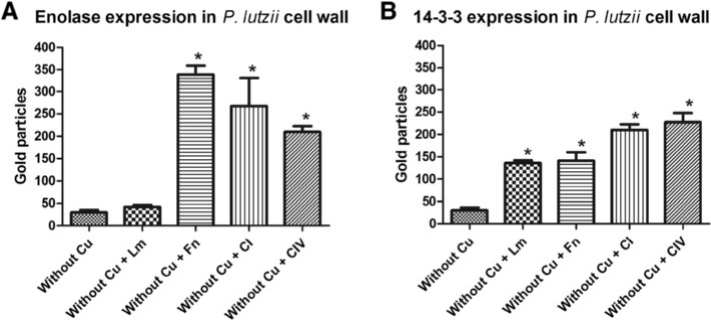
**Quantitative analyses of expressed A) enolase and B) 14-3-3 proteins during the interaction with ECM components.**

Real-time PCR analysis revealed higher expression levels of two adhesins, enolase and 14-3-3, under conditions of Cu depletion and in the presence of the different ECM components. Thus, we explored the subcellular location of these proteins using anti-enolase and anti-14-3-3 polyclonal antibodies in combination with immunoelectron microscopy (IEM) to confirm the differential expression of these proteins during the interaction of *P. lutzii* with the different ECM components (Figures 3 and 4). *P. lutzii* yeast cells with and without Cu in contact with all the ECM components were processed by post embedding with gold particles. The immunocytochemistry assays revealed a ubiquitous distribution of the gold particles in all conditions, but we observed that, when the fungus was grown under simulated infection conditions, there was an increased expression of enolase and 14-3-3 (indicated with arrows in Figures 3 and 4) in the fungal cell wall. A quantitative analysis was developed by counting the number of expressed enolase and 14-3-3 proteins in the fungal cell wall and a real increase of protein expression occurred in the cell wall during its interaction with all ECM components (Figure 5). These results showed that these proteins, when interacting with the host structures, are recruited to the cell wall and may be used during this interaction, as shown by da Silva et al. [26] and Marcos et al. [27].

### *Paracoccidioides lutzii* protein expression identification by mass spectrometry during Cu deprivation in the presence of different ECM components

**Figure 6:**
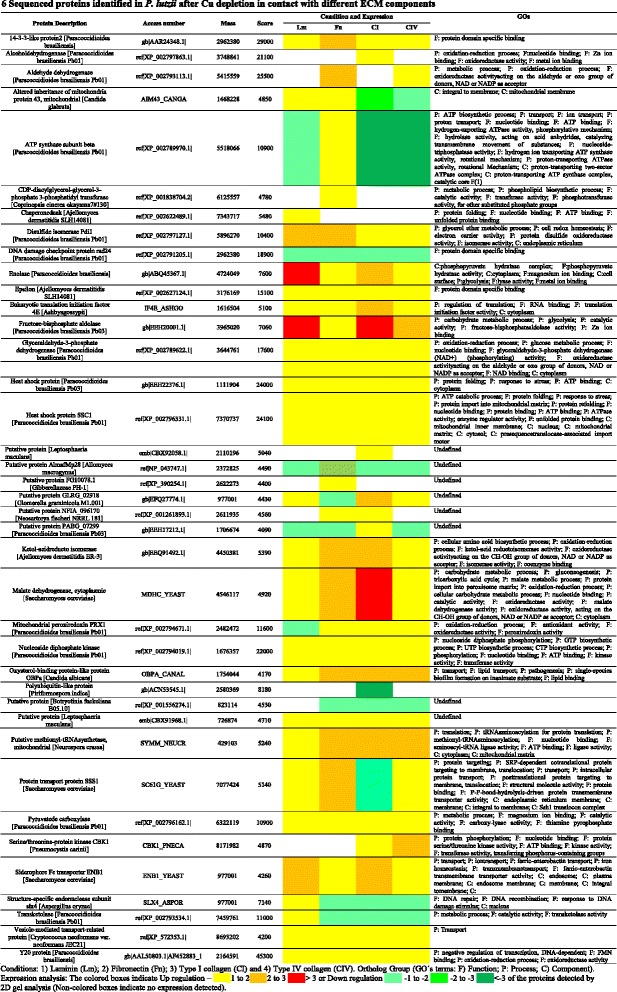
**Sequenced proteins identified in** ***P. lutzii*** **after Cu depletion in contact with different ECM components.**

**Figure 7:**
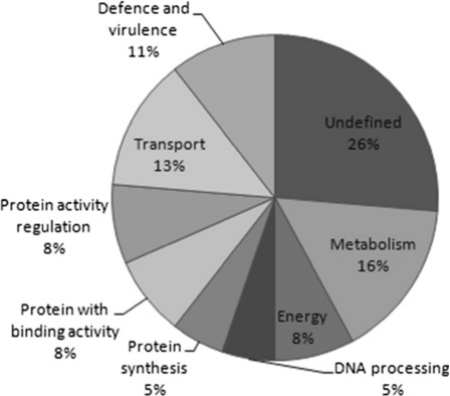
**Classification of the function of the proteins identified by MS.**

**Figure 8:**
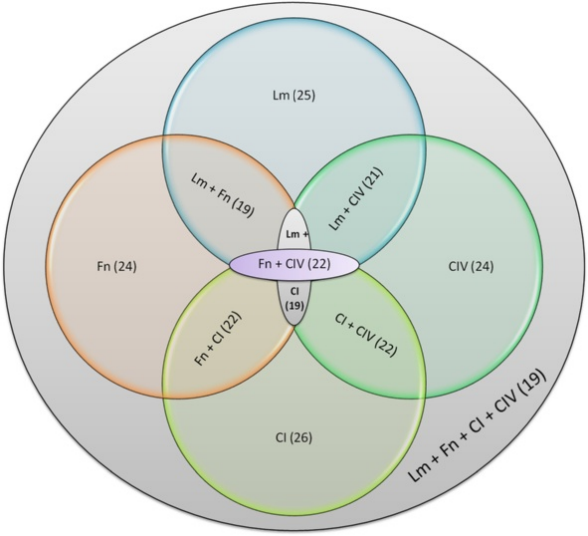
**Proteins identified by Venn diagram, which interact with the different ECM components: overview of the number of the proteins differentially expressed by** ***P. lutzii*** **during its interaction with the host components.**

The protein profile of *P. lutzii* grown in an environment depleted of Cu was used as a master gel for comparison after contact with the ECM components (laminin, fibronectin, type I and type IV collagen). Based on these analyses, differentially expressed proteins were selected for identification by mass spectrometry (MS), as well as the results of the expression analysis. The results indicate that the fungus uses an arsenal of proteins during its interaction with the host ECM components (Figure 6). This arsenal is composed of proteins that are related to different cell processes (Figure 7) and an extensive number of proteins that may be involved with the interaction between the fungus and the host. Additionally, some of the identified proteins are involved in metabolism and the transport of micronutrients, such as Fe and Zinc (Zn), revealing the adaptation of the fungi to the environment. We also observed proteins that are involved in DNA repair and heat shock proteins that are induced as a response to the stress that this fungus was submitted to during Cu starvation. The contact of the fungus with different ECM components led to the different responses, forcing the pathogen to adapt to the environment. The results found during this interaction with each of the different ECM components vary and these proteins can be shared to interact with the different components. We observed that 19 proteins are shared during the interaction with all ECM components, while 21 are common between the interaction of *P. lutzzi* with Lm and Fn, 19 with Lm and CI, 21 with Lm and CIV, 22 with Fn and CI, 22 with Fn and CIV and 22 with CI and CIV, as summarized in Figure 8. Comparative proteomic analysis with *P. lutzii* maintained in a medium supplemented with Cu warrants future study.

### Real-time PCR to validate 2D-E assay results

**Figure 9:**
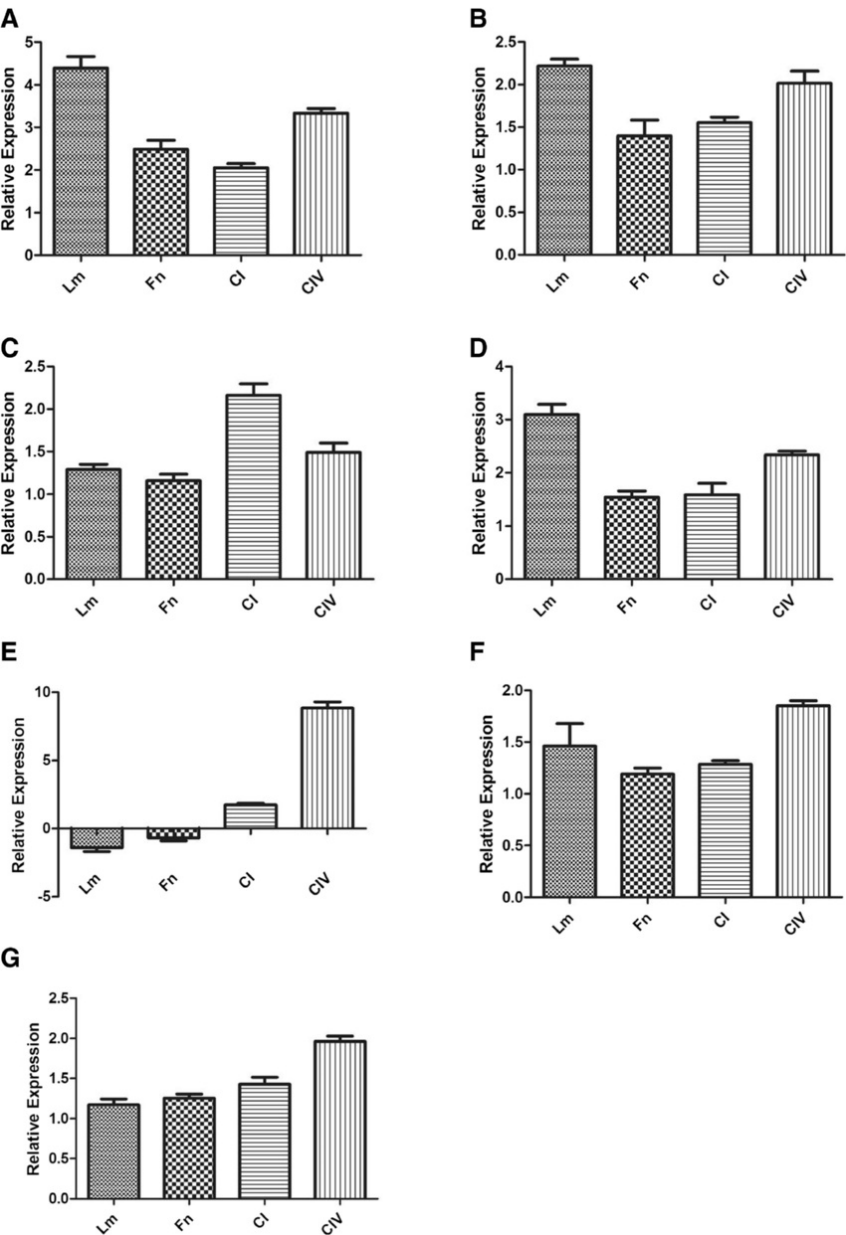
**Quantification of differentially expressed genes and proteins without Cu in contact with the different ECM components using Real Time PCR. A)** Enolase; **B)** 14-3-3; **C)** Aldolase; **D)** GAPDH; **E)** Serine/threonine-protein kinase CBK1; **F)** Siderophore Fe transporter ENB1; **G)** Vesicle-mediated transport-related protein.

Six of the proteins identified when using the proteomic approach were chosen to validate the regulation of gene expression by real-time PCR. The chosen genes for this evaluation were enolase, 14-3-3, aldolase, GAPDH, serine/threonine-protein kinase CBK1, siderophore-Fe transporter ENB1 and vesicle-mediated transport-related protein. These genes were selected based on their importance in interactions between *Paracoccidioides* and the host. This analysis confirmed a regulation of the expression of the proteins identified using proteomics and showed that Cu depletion led to a regulation at the transcriptional levels of the proteins, since the observed gene expression levels were totally correspondent with the protein expression levels (Figure 9). Table 1 summarizes these results.

**Table 1:** **Comparison on the expression levels of the identified proteins and its coding genes**

	**Proteomics**				**Real-time PCR**			
	**Lm**	**Fn**	**CI**	**CIV**	**Lm**	**Fn**	**CI**	**CIV**
**ENO**	↑↑↑	↑	↑	↑↑	↑↑↑	↑	↑↑	↑
**14-3-3**	↑↑	↑	↑	↑	↑	↑↑	↑	↑
**ALD**	↑	↑	↑↑	↑	↑↑↑	↑	↑↑↑	↑↑
**GAPDH**	↑↑↑	↑	↑	↑↑	↑	↑	↑	↑
**CBK1**			↑	↑↑	↓	↓	↑	↑↑↑
**ENB1**	↑↑	↑	↑↑	↑	↑	↑	↑	↑
**VES**	↑	↑	↑	↑	↑	↑	↑	↑↑

Upregulation is represented by ↑ and downregulation is represented by ↓ and the number of arrows indicates the identified levels of expression (1 arrow - 1 to 2, 2 arrows - 2 to 3 and 3 arrows - 3 or more or less folds expressed when compared with the control).

### *In silico* analysis of putative proteins identified in 2D gel analysis

We observed 8 proteins that had unknown functions and made functional inferences using *in silico* analysis. It is known that surface proteins are important for *Paracoccidioides* pathogenesis, so we performed *in silico* analysis to identify adhesin-like proteins using FaaPred software, which predicts fungal adhesins and adhesin-like proteins. This analysis predicted 2 proteins as adhesin-like proteins (Table 2), revealing interesting candidates for further studies of the protein relationships with *Paracoccidioides* virulence.

**Table 2:** ***In silico*** **analysis of sequenced putative proteins using the software FaaPred**

**Protein**	**Access number**	**Prediction**	**SVM Score**
Putative protein [*Leptosphaeria maculans*]	emb|CBX92058.1|	Adhesin	0.33534569
Putative protein AlmafMp28 [*Allomyces macrogynus*]	ref|NP_043747.1|	Non-adhesin	−1.7894963
Putative protein FG10078.1 [*Gibberellazeae* PH-1]	ref|XP_390254.1|	Non-adhesin	−1.0139011
Putative protein GLRG_02918 [*Glomerella graminicola* M1.001]	gb|EFQ27774.1|	Adhesin	−0.63979878
Putative protein NFIA_096170 [*Neosartorya fischeri* NRRL 181]	ref|XP_001261893.1|	Non-adhesin	−1.5268407
Putative protein PABG_07299 [*Paracoccidioides brasiliensis* Pb03]	gb|EEH17212.1|	Non-adhesin	−1.3347091
Putative protein [*Botryotinia fuckeliana* B05.10]	ref|XP_001556274.1|	Non-adhesin	−2.6864038
Putative protein [*Leptosphaeria maculans*]	emb|CBX91968.1|	Non-Adhesin	−1.2838827

**Figure 10:**
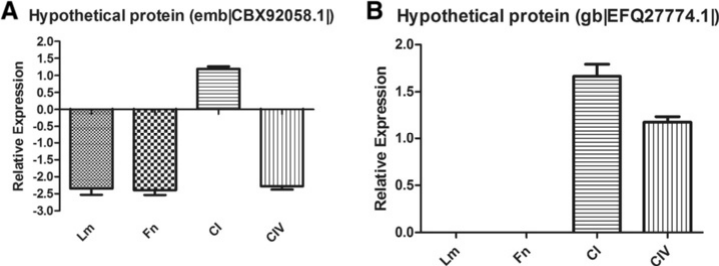
**Quantification of predicted adhesins differentially expressed without Cu in contact with the different ECM components using Real Time PCR. A)** Putative protein [Leptosphaeria maculans] (emb|CBX92058.1|), **B)** Putative protein GLRG_02918 [Glomerella graminicola M1.001] (gb|EFQ27774.1|).

The expression of these adhesin-like proteins was evaluated using real-time PCR during the interaction of *P. lutzii* with the host ECM components (Figure 10). These results demonstrated that proteomic assays could identify unknown molecules that may be related to the adhesion of *Paracoccidioides.* These results allow for the identification of some adhesin-like proteins that may be important for fungal adhesion to different ECM components in different sites of the host organism and determining the contributions of these proteins to fungal establishment.

## Discussion

All organisms most likely depend on efficient metal transport systems for survival. Fe and Cu are particularly important metals because they participate in vital reactions and are both cofactors of many metabolic enzymes and are essential nutrients for the maintenance of life [28]. In excess, these metals can be toxic; however, disturbances to the Fe and Cu levels can have serious effects on cellular metabolism, growth and development [29]. Cu exists as a trace element, and is found in low concentrations in living organisms. However, Cu is an important catalytic cofactor that guides several important biological processes that are essential for life. Cu regulates changes in protein structure, catalytic activity and protein-protein interactions. In this manner, it controls a varied series of biochemical events; Cu may modulate critical fungal virulence factors [9].

The importance of our work lies precisely in the fact that *Paracoccidioides* is a facultative intracellular pathogen. Because of this characteristic, any external changes in the intracellular environment can directly influence the pathogenicity of this organism. Additionally, the period evaluated and ECM components used in this work (laminin, fibronectin and collagen) are essential in the initial interaction of the pathogen with the host, thus any change of homeostasis (such as deprivation of micronutrients) would have a direct impact on this initial interaction and, consequently, virulence.

In our study, the depletion of Cu altered the adhesion pattern of *Paracoccidioides* to ECM components. There was a significant (p < 0.05) increase in adhesion to all ECM components, which was different from what we observed for Fe. Fe depletion led to a significant decrease in adhesion, corroborating the results of Parente *et al*. [17], who demonstrated that Fe supplementation increased the susceptibility of mice to *Paracoccidioides* infection. Using real-time PCR, we observed that the depletion of these metals altered the expression patterns of adhesins, which are important components in the *Paracocccidioides*-host interaction. When the fungus was grown in Cu-depleted conditions, overexpression of genes encoding adhesins was observed, whereas down-regulation of the same genes was observed during Fe-depletion. Complementing these results by using immunogold analysis, we clearly observed an increased expression of two important adhesins, enolase and 14-3-3 protein, when the fungus was in an environment depleted of Cu and in contact with different ECM components. In addition, we observed that this increase occurred in the fungus cell wall, suggesting that the intensification of adhesion may be attributable to the great quantity of these adhesins in the fungal cell wall.

The identification of proteins involved in the initial phase of host-fungus interactions is very important and can result in the identification of potential drug targets for future therapeutic research to prevent the early infectious process. The 2-DE technique was used in this study to determine proteins that may be involved in the interaction among *Paracoccidioides* and ECM components when the fungus was deprived of Cu, leading to an increase in the adhesion of the fungi.

Our goal with the proteome was to understand what makes the fungus increase its capacity to adhere to all ECM components after Cu deprivation, which is why our main control was only the fungi maintained without Cu. All of the proteins identified in these results are expressed during Cu depletion, but they suffer modifications in expression because of the contact of the fungi with the host.

Our proteomic assays identified 39 proteins expressed by the fungi after Cu depletion and during its interaction with the ECM components. These results contribute to our knowledge of the molecular arsenal used by *Paracoccidioides* to adhere, invade and cause systemic mycosis.

Among the identified proteins, some of them were already identified as being important to the interaction of *Paracoccidioides* spp. with the host and could be associated with dissemination of the infection. These proteins are 14-3-3, enolase, aldolase and glyceraldehyde-3-phosphate dehydrogenase; all of which are currently described as adhesins [26,27,30-32]. These adhesins have their expression up-regulated while interacting with the host with all ECM components. These results, once again, reveal the importance of these molecules in the interaction of *P. lutzii* with the host. The experiment indicates that, during Cu starvation, these molecules are maintained at basal levels of expression, but contact with the host signaled the fungus to increase the expression of these proteins to adhere to the host and, from the host, obtain the necessary Cu for maintenance. With these results, we can understand how the fungus uses its protein arsenal to adapt to the host and have success during the infection process.

Increased enolase expression was verified; this protein was recently described as a fibronectin ligand related to *Paracoccidioides* adhesion and is considered a virulence factor [31]. Recent studies have shown that enolase has different distributions and biological functions [33] and is expressed at different sites in eukaryotes and prokaryotes with distinct biological functions, such as the laminin ligand in *Staphylococcus aureus* [34] and enolase binding of human plasminogen in *Trichomonas vaginalis* [35], *Streptococcus pneumoniae* [36], *Leishmania mexicana* [37] and *Fasciola hepatica* [38]. In our study, enolase was differentially expressed when the fungus was in contact with all of the ECM components, not only fibronectin, as previously described by Donofrio *et al.* [30]. However, it is worth noting that the strain used in the latter work was a *Paracoccidioides brasiliensis* strain named Pb18, which could be an important factor for further investigation [31]. Nevertheless, data from Nogueira *et al.* [20] also demonstrated binding of enolase to laminin and type I collagen, corroborating the findings of this study. Moreover, data from Nogueira *et al.* [20] also indicates that enolase is an important *Paracoccidioides* molecule used during its interaction with the host. The expression of this protein in this present study corroborates earlier data suggesting that *Paracoccidioides* enolase can be used to adhere to and, perhaps, invade host cells through an interaction with human plasminogen [31,33,36,38]. The ability to bind plasminogen has been associated with invasive properties in pathogenic microorganisms [37,39,40] and can facilitate their penetration to the basement membrane and permit associations with fibronectin and laminin. Although *Paracoccidioides* is considered a facultative intracellular pathogen, fibronectin adhesin ligands can also mediate cellular invasion. Our electron microscopy results demonstrated an increase of enolase during the interaction between the fungus cell and the ECM components, mainly at the cell wall. These current results corroborate those of Marcos *et al.* [27], who reported a substantial increase of enolase in the cell wall during infection of pneumocytes (A549 cells), suggesting the importance of this protein in fungal adhesion to the host [41].

We also observed the overexpression of the 14-3-3 protein when the fungus was in contact with all of the ECM components studied. The 14-3-3 proteins are a family of highly conserved acidic dimeric proteins that have been implicated in a variety of cellular processes in eukaryotes [41-44]. In *Paracoccidioides,* a 30-kDa protein has been identified and characterized as a 14-3-3 protein that causes structural modification of polymerized actin microfilaments and cytokeratin, induces apoptosis when interacting with epithelial cells and is capable of binding to laminin [45,46]. In addition, da Silva *et al.* [26] demonstrated that, during when *Paracoccidioides* interacted with A549 cells, an apparent increase of the 14-3-3 protein on the cell wall of the fungus occurs, suggesting that this protein may be involved in host-parasite interactions.

Glyceraldehyde-3-phosphate dehydrogenase (GAPDH) and fructose 1,6-biphosphate aldolase (FBA) (part of the glycolytic pathway) were observed to be overexpressed during the interaction of *Paracoccidioides* with all of the ECM components studied. Barbosa *et al.* [31] demonstrated that GAPDH from *Paracoccidioides* is able to bind to laminin, fibronectin and type IV collagen and the inhibition of this protein causes decreased adhesion rates of the fungus to epithelial cells [31]. FBA appears to be important in host-parasite interactions [47,48]. A FBA homologue was previously described as an immunogenic protein of *Paracoccidioides* [18]. Additionally, this fungus contains two genes encoding two different Class II FBAs. Phylogenetic analysis supports the concept of gene duplication for FBA genes, constituting a two-member family whose function could differ in the fungal cells. In addition, expression analysis performed using northern blot and RT-PCR indicated a differential expression for Pbfba1 and Pbfba2 in *Paracoccidioides* cells, suggesting distinct functions for both proteins [32]. An interesting and unsuspected feature observed during this present work is that both *Paracoccidioides* FBAs appeared to play roles in the fungal interaction with the host. PbFBA1 has immunogenic properties, as indicated by the fact that the native protein was recognized by the sera of infected patients [18].

Under conditions of Cu depletion, we observed an increase in the expression of ENB1, the enterobactin Fe transporter, a high-affinity siderophore in microbial systems. This increase may have occurred because most Fe-acquisition systems are Cu dependent, and Cu deficiency may force the fungi to use the siderophore system to acquire Fe from the culture media [49]. The siderophores are generally used by microorganisms in situations of low Fe concentrations. However, Froissard *et al.* [49] reported that ENB1 appears to be constitutively expressed in the plasma membrane of *S. cerevisiae*, and its expression is not affected by the substrate concentration.

Another protein that exhibited increased expression was CBK1, a serine/threonine protein kinase of the RAM signaling network. Cbk1p is similar to the human myotonic dystrophy kinase and is essential for normal morphogenesis in *S. cerevisiae*. It is involved in regulating cellular morphogenesis, polarized growth, and septum destruction. Cbk1p activity is regulated by both phosphorylation and specific localization; the protein relocates to the cytoplasm upon DNA replication stress [50]. Cbk1 has been previously implicated in regulating polarized morphogenesis, gene expression and cell integrity in *S. cerevisiae* and is also critical for heat shock and cell wall stress signaling. These results obtained using *S. cerevisiae* constitute the first evidence that Cbk1 kinase regulates MAPK-dependent stress signaling and provides mechanistic insight into Sdp1 phosphatase regulation via Bck2, a protein associated with the Pkc1-Mpk1 cell integrity pathway [51]. In *C. albicans,* Cbk1 is a daughter cell protein involved in cell wall degradation from the daughter side during cell separation [52]; recently, this protein was observed to be involved in *C. albicans* biofilm formation [53]. In this current work, we verified that Cu depletion promotes stress and cellular changes in the cell wall composition of *Paracoccidioides*. In addition, the increased expression of all previously described *Paracoccidioides* adhesins and the increased expression of kinases may suggest that remodeling of the *Paracoccidioides* cell wall could occur through activation of the RAM pathway. This mechanism should be investigated in future studies.

The identification of a vesicle-mediated transport-related protein overexpressed during the interaction of *Paracoccidioides* with all of the ECM components is an interesting finding because Vallejo *et al.* [54] recently described the presence of these structures in *Paracoccidioides,* including antigenic molecules, that are recognized by total sera from PCM patients. Vallejo *et al.* [22] also described the proteomic analysis of extracellular vesicles and vesicle-free released proteins in *Paracoccidioides* and demonstrated that these vesicles carry different molecules, including virulence factors previously described for *Paracoccidioides.* It is important to emphasize that we observed molecules such as GAPDH, enolase, aldolase, 14-3-3 and malate dehydrogenase in the current study [23].

In our proteomic results, we also observed several putative proteins with currently unknown functions. Therefore, an *in silico* analysis was performed to identify sequences that may play important roles as adhesins during the *Paracoccidioides*-host interaction. For this purpose, the FaaPred software [55] was used to predict fungal adhesins and adhesin-like proteins. In our analysis, we predicted 2 proteins that might be adhesin-like proteins. Although these are preliminary results, this type of analysis demonstrates that a considerable portion of the arsenal of molecules used by *Paracoccidioides* while interacting with the host might still be unknown. These findings promote the need for further studies characterizing new virulence factors of this fungus.

## Conclusions

Understanding the adaptive response to different growth conditions, elucidating the processes of adhesion and cell invasion, and identifying the genes that are differentially expressed during the parasite-host interaction may help elucidate the mechanisms used for survival and growth of *Paracoccidioides* in various human tissues. In our study, we observed that proteins related to virulence were expressed against all ECM components, which might explain the increase in fungal adhesion. The fungus-host interaction includes a complex network of signaling pathways that are interconnected with regards to protein expression in a given situation or set of conditions. One goal of this study was to identify the differentially expressed proteins of *Paracoccidioides* when in contact with extracellular matrix components. In this sense, the 2-DE technique was extremely useful and provided an overview of the cellular components at the particular moment of contact.

## Methods

### Ethics statement

The 14-3-3 and enolase rabbit preimmune serum used in this study was obtained from two previous studies, Silva et al. [26] and Donofrio et al. [31] with the approval of the Ethics Committee on Animal Experiments of the Faculty of Pharmaceutical Sciences of Araraquara – UNESP, Processes Protocols 10/2011/CEUA/FCF and 08/2001/CEUA/FCF respectively. The experiments in these previous studies were performed in strict accordance with Brazilian Federal Law 11,794 that established procedures for the scientific use of animals and the state law establishing the Animal Protection Code of the State of São Paulo. All efforts were made to minimize suffering by the animals used in the current study.

### Strain and culture conditions

The *Paracoccidioides lutzii* 01 strain (ATCC MYA-826) was used in all experiments. The yeast phase was maintained *in vitro* in Fava-Netto’s medium [56] for 7 days at 36°C. For the adhesion assay, the fungus was incubated under three conditions: (1) complete McVeigh-Morton (MVM) medium [57,58] containing glucose 1%, KH_2_PO_4_ 11 mM, MgSO_4_.7H_2_0 2 mM, CaCl_2_.2H_2_0 1 mM, (NH_4_)_2_SO_4_ 15 mM, L-Asparagine 0.02%, L-Cystine 0.002%, vitamin supplements 1%, and trace element supplement 0.1%, (2) MVM without Cu reagent (MVM-W-Cu) and (3) MVM without Fe reagent. Moreover, bathocuproinedisulfonic acid (BCS) (Sigma-Aldrich, St. Louis, MO, USA) and bathophenanthroline disulfate (BPS) (Sigma-Aldrich, St. Louis, MO, USA) were used to chelate the Cu and Fe ions, respectively. The fungus maintained in Fava-Netto’s medium was transferred to the three conditions described above and incubated for 3 h at 37°C.

### Fungal adherence assays

ECM proteins, represented by laminin, fibronectin, and types I and IV collagen (Sigma-Aldrich, St. Louis, MO, USA), were immobilized on 24-well microtiter plates at 50 μg/ml diluted in carbonate-bicarbonate buffer (pH 9.6). The plates were incubated for 1 h at 25°C, incubated overnight at 4°C and washed with PBS. The fungal suspensions were washed again and suspended in PBS. Suspensions (500 μL) of yeast containing 10^6^ cells/ml (0.5 OD at 550 nm) were transferred to microtiter plates containing ECM proteins and incubated for 2 h at 37°C. The wells were washed three times with PBS, and trypsin was used to recover the cells, which were suspended in 500 μL of FACSFlow® (BD, Becton Dickinson Company) and then examined by flow cytometry. All flow cytometry analyses were performed using a BD FACS Canto. The data (number of cells) were analyzed using BDFACS Diva software. The adhesion experiments were made at three independent times, in triplicate. As an experimental control, wells without ECM were inoculated with the fungal suspension and adhesion to the plate was analyzed. The adhesion values found in this control were subtracted from the adhesion values found in the tests with the different ECMs.

### Expression analysis of enolase and 14-3-3 proteins in *Paracoccidioides lutzii* yeast cells when interacting with ECM components after micronutrient depletion using immunogold labeling

To examine the expression of the enolase and 14-3-3 proteins of *P. lutzii*, we performed immunocytochemistry at the ultra-structural level using immunogold labeling. For each experiment, a suspension of *P. lutzii* yeast cells without Cu and *P. lutzii* without Cu in the presence of ECM components (10^8^ cells/ml) were fixed (2.5% v/v glutaraldehyde in 0.1 M sodium cacodylate buffer, pH 7.2) for 24 h at 4°C and sent to the electron microscopy service of the Institute of Biomedical Sciences (ICB-I) USP-SP for preparation of ultrathin sections. After fixation, the cells were rinsed several times using the same buffer, and free aldehyde groups were quenched with 50 mM ammonium chloride for 1 h, followed by block staining in a solution containing 2% (w/v) uranyl acetate in 15% (v/v) acetone for 2 h at 4°C. The material was dehydrated in a series of ascending concentrations of acetone (30 to 100% v/v) and embedded in LR Gold resin (Electron Microscopy Sciences, Washington, Pa.). The ultrathin sections were collected on nickel grids, preincubated in 10 mM PBS containing 1.5% (w/v) bovine serum albumin (BSA) and 0.05% (v/v) Tween 20 (PBS-BSA-T). The sections were subsequently incubated overnight with a polyclonal antibody against the enolase and 14-3-3 proteins (diluted 1:50). After washing with PBS-BSA-T, the grids were incubated overnight with the labeled secondary antibody (rabbit IgG, Au conjugated, 10 nm; diluted 1:10). Controls were incubated with rabbit pre-immune serum at 1:50, followed by incubation with the labeled secondary antibody. After incubation, the grids were washed with the buffer described above, followed by a wash with distilled water, and staining with 3% uranyl acetate (w/v) and 4% lead citrate (w/v). Finally, the grids were observed using a Jeol 1010 transmission electron microscope (Jeol, Tokyo, Japan). Using the software, Image Tool v. 3.00 [59], 10 different cells of each tested situation were selected and the proteins marked with gold particles were counted, providing quantitative analyses of the expression of this protein when interacting with the ECM components after Cu depletion.

### RNA isolation and cDNA synthesis

Total RNA was extracted using the following conditions: (1) *P. lutzii* yeast cells maintained in MVM without Cu; and (2) yeast cells cultivated in MVM without Cu, incubated for 3 h at 37°C followed by the addition of the different ECM components (laminin, fibronectin, and types I and IV collagen) at 50 μg/ml, and incubated for an additional 2 h at 37°C. These preps were used for the Real Time PCR assays.

Total RNA was extracted from all experimental conditions using Trizol reagent (Invitrogen Life Technologies, Carlsbad, CA). First-strand cDNA synthesis was performed using reverse transcriptase (RevertAid™ H Minus Reverse Transcriptase, Fermentas Life Sciences, Canada) and 1 μg of total RNA. First-strand cDNA was used as a template to synthesize the second-strand of cDNA using the SMART PCR cDNA synthesis kit (Clontech Laboratories, Palo Alto, CA, USA).

### Proteomic analysis of *Paracoccidioides lutzii* protein expression during Cu deprivation in the presence of different ECM components

The total protein extracts of *P. lutzii* were obtained in the yeast phase under different conditions, as shown in Table 3, to isolate and characterize the proteins differentially expressed in the different conditions.

**Table 3:** **Conditions** ***of P. lutzii*** **tested by proteomic assays**

	**Condition**
0	*P. lutzii* maintained in MVM without Cu
1	*P. lutzii* maintained in MVM without Cu + laminin
2	*P. lutzii* maintained in MVM without Cu + fibronectin
3	*P. lutzii* maintained in MVM without Cu + type I collagen
4	*P. lutzii* maintained in MVM without Cu + type IV collagen

Cells from *P. lutzii* in the yeast phase submitted to the different conditions (Table 3) were centrifuged (5000 *g* for 10 min) and washed 3–5 times with cold water to remove the culture medium. Then, 10 mM Tris – HCl and protease inhibitors (1 mM pepstatin, 1 mM leupeptin, 1 mM aprotin, 1 mM antipain, 1 mM chymostatin and 1 mM PMSF) were added to the pellet, which was subsequently homogenized with liquid nitrogen, macerated with glass beads and vortexed for 30 min. This preparation was then centrifuged for 45 min at 13000 *g*, and the supernatant collected. The protein concentration of the extracts was quantified using the Bradford Assay [60] (Bio-Rad Laboratories, California, USA). The samples were then analyzed by SDS-PAGE. The protein components of the extracts were subjected to isoelectric focusing using EttanIPGphor 3 (GE Healthcare, Buckinghamshire, UK). The second dimension, performed to separate proteins according to molecular weight, was conducted in a 12.5% polyacrylamide gel according to Laemmli et al. [61]. The gels were stained with Coomassie Brilliant Blue G-350 [62]. The data analysis was performed using Image Master 2D Platinum software (GE Healthcare, Buckinghamshire, UK) to compare the protein profile of the *P. lutzii* depleted of Cu before and after the fungus came into contact with the different ECM components. The 2D Platinum software analyzes the volumes of the spots and determines how much the expression of the selected spots were increased or decreased when compared with the control (*P. lutzii* without Cu) and our tests (*P. lutzii* without Cu in contact with the different ECM components)”.

### Protein identification by mass spectrometry (MS)

Protein spots were excised from 2-DE gels, cut, distained, reduced, alkylated and subjected to tryptic digestion using 10 ng/ml of Trypsin Gold (Promega Corporation, Wisconsin, USA), according to Celedon *et al.* [63]. After digestion, the peptides were extracted twice with [50 μL of 60% (v/v) methanol, 1% (v/v) formic acid (FA)], twice with [50 μL of 50% (v/v) acetonitrile (ACN) and MS-grade-water, 1% (v/v) FA] and once with 50 μL of ACN (100%). All supernatants were combined and vacuum dried. Peptides were then suspended in 13 μL of 0.1% (v/v) FA for MS analysis. Peptide masses were measured using a nanoelectrospray ionization quadrupole time-of-flight hybrid mass spectrometer (Q-TOF Ultima; Waters) coupled to a nano-HPLC (Cap-LC, Waters), as described by Fiorani Celedon *et al.* [63]. The resulting spectra were processed using ProteinLynx v.4.0 software (Waters) and MASCOT MS/MS Ion Search (http://www.matrixscience.com). The sequences were compared with those in the NCBI and SwissProt databanks.

### Real-time PCR

Real-time PCR analysis was used for three different purposes in this study:

1. to evaluate the adhesin gene expression during contact between *P. lutzii* and the different ECM in Cu-depleted conditions. For this, we evaluated the gene expression of 6 known adhesins: enolase (ENO), Glyceraldehyde-3-phosphate dehydrogenase (GAPDH), GP43, malate synthase (MLT), triosephosphate isomerase (TPI), and 14-3-3;
2. to confirm the differential expression of the proteins found in the proteomics assay. To do so, we compared the cDNA of *P. lutzii* grown in Cu-depleted conditions with the cDNA of *P. lutzii* grown in Cu-depleted conditions but in contact with one of the four ECM components. We used 12 different primers: cell surface protein (CS protein), cytokine-inducing glycoprotein (CIGI), ENO, glucose-6-phosphate dehydrogenase (G6PD), glutamine synthetase (GSA), RDS1 protein (RDS1), 14-3-3, aldolase (ALD), GAPDH, serine/threonine-protein kinase CBK1 (CBK1), siderophore Fe transporter (ENB1), and vesicle-mediated transport-related protein (VES);
3. to evaluate the expression of the adhesin-like proteins identified in the proteomics assay. We used 2 primers for this purpose: emb|CBX92058.1| and gb|EFQ27774.1|. The SIGLA used to name the primers corresponded to the accession number of the studied gene.

The reaction mixtures contained 2 μL of cDNA (40 ng), 12.5 μL of Maxima® SYBR Green/ROX qPCR Master Mix (2×) (Thermo Fisher Scientific, Massachusetts, USA), and 0.5 μM of forward and reverse primers. The volume was brought to 25 μL with nuclease-free water. The reaction program was as follows: 50°C for 2 min, 95°C for 10 min, 40 cycles of 95°C for 15 s, and a period of annealing and synthesis at 60°C for 1 min. Following the PCR, a melting-curve analysis was performed, which confirmed that the signal corresponded to a single PCR product. The reactions were performed in an Applied Biosystems 7500 cycler. The data were analyzed using the 2^−ΔΔCT^ method. The cycle threshold values for the duplicate PCRs for each RNA sample were averaged, and then the 2^−ΔΔCT^ values were calculated. The constitutive gene encoding the 60S ribosomal L34 was used as the endogenous control. A negative-control sample containing all reagents except *P. lutzii* cDNA was used. After 40 rounds of amplification, no PCR products were detected in this reaction. These experimental results represent numbers of three independent experiments in triplicate.

All of the primer sequences used in the real-time PCR are listed in Additional file 1: Table S1.

### *In silico* analysis of putative proteins identified in 2D-E analysis for adhesin-like protein predictions

To predict adhesin-like proteins, we used the software, FaaPred (Fungal Adhesins and Adhesin-like proteins predictions), developed by Ramana and Gupta [55]; this software uses an SVM-based method (Support Vector Machine) to identify fungal adhesins available on-line (http://bioinfo.icgeb.res.in/faap/). This software searches for N-terminal carbohydrates or peptide-binding domains, central Ser-and Thr-rich glycosylated domains and C-terminal regions that mediate covalent cross-linking to the fungal cell wall through modified glycosyl phosphatidyl inositol (GPI).

### Statistical analyses

All statistical analyses were performed using one-way ANOVA with Tukey’s coefficient. The results of the statistical analyses were considered significant when the *p* value was <0.05. These analyses and graphs were made using Prism 5 (GraphPad Software Inc.).

## Additional file

Additional file 1: Table S1 Primers used for the real-time PCR assays.
