# Supplementary material for: Alterations of protein expression in conditions of copper-deprivation for Paracoccidioides lutzii in the presence of extracellular matrix components
Source: BMC Microbiol. 2014 Dec 13;14:302. doi: 10.1186/s12866-014-0302-7 (PMC4302596; doi:10.1186/s12866-014-0302-7)
Supplement: Additional file 1: Table S1 — Primers used for the real-time PCR assays. [file 12866_2014_302_MOESM1_ESM.doc]

**Supplementary material**

**Table S1.** Primers used for the real-time PCR assays.

| **Gene** | **Primers** |
| --- | --- |
| Enolase | Sense 5´-TAGGCACCCTCACTGAATCC-3´  Antisense 5´-GCTCTCAATCCCACAACGAT-3´ |
| GADPH | Sense 5´-AAATGCTGTTGAGCACGATG-3´  Antisense 5´-CTGTGCTGGATATCGCCTTT-3´ |
| GP43 | Sense 5´-CTTGTCTGGGCCAAAAACTC-3´  Antisense 5´-GCCAGGGTTTGTTTGACTGT-3´ |
| Malate syntethase | Sense 5´-GTTCCCTTCATGGATGCCTA-3´  Antisense 5´-TCTTTGATGGGGATTTGAGC-3´ |
| Triosephosphato isomerase | Sense 5´-CCTTACGGCAGAATGACGTT-3´  Antisense 5´-GCCATTTCCATGTCAGGTCT-3´ |
| 14-3-3 | Sense 5´ -GTTCGCTCTTGGAGACAAGC-3´  Antisense 5´ -AGCAACCTCAGTTGCGTTCT-3´ |
| Aldolase | Sense 5´-CAAGGCCGAGTTCAAAGAAG-3´  Antisense 5´-GGATTCCCAACAGCACTCAT-3´ |
| CBK1 | Sense 5´ -TTCGTGCTGTGAATCTCGAC-3´  Antisense 5´ -AAATTGGCTGGGGTCTCTTT-3´ |
| ENB1 | Sense 5´ -TTCGCAGTAAACGTGCAGTC-3´  Antisense 5´ -CGTCGTGTCAGCGATAAAGA-3´ |
| VES | Sense 5´ -AACACCGTGAAGGTTTGGAG-3´  Antisense 5´-GAGCAGCATTTTCGAGTTCC-3´ |
| emb|CBX92058.1| | Sense 5´ -CTTGACACGGTAGGGTCGTT-3´  Antisense 5´ -CCGCTTTGTTGGTAACGAAT-3´ |
| gb|EFQ27774.1| | Sense 5´ -TGCGGATAGTTCATCAGTCG-3´  Antisense 5´ -TGGGTGATATCAGCACAGGA |
| L34 (endogenous gene) | Sense 5´-TGTCTACACTGCGCAAGGAC-3´  Antisense 5´-ATGTGTTGGTGGGAGAGGAG-3´ |
